# Correlation between dietary factors and Parkinson’s disease revealed by the analysis of Mendelian randomization

**DOI:** 10.3389/fnut.2024.1273874

**Published:** 2024-05-22

**Authors:** Shan Zeng, Aishanjiang Yusufujiang, Chunli Zhang, Chen Yang, Hongyan Li

**Affiliations:** ^1^Department of Graduate School, Xinjiang Medical University, Urumqi, Xinjiang, China; ^2^Department of Neurology, People’s Hospital of Xinjiang Uygur Autonomous Region, Urumqi, China

**Keywords:** Parkinson’s disease, Mendelian randomization, dietary factors, gut microbiome, *Parabacteroides goldsteinii*

## Abstract

**Background:**

The intricate interplay between dietary habits and the development of Parkinson’s Disease (PD) has long been a subject of scientific inquiry. Mendelian Randomization (MR) emerges as a potent tool, harnessing genetic variants to infer causality in observational data. While evidence links diet to Parkinson’s Disease (PD) etiology, a thorough MR exploration of dietary impacts on PD, particularly involving gut microbiota, is still emerging.

**Methods:**

This research leverages the IEU Open GWAS project’s vast GWAS database^1^ to address the knowledge gap in understanding diet’s influence on PD, employing a diverse range of dietary variables. Our holistic dataset includes various foods like processed fava beans, bap, red wine, to cheese, reflecting a commitment to untangling dietary complexities in PD etiology. Advancing from initial dietary-PD associations, we innovatively explore the gut microbiota, focusing on *Parabacteroides goldsteinii,* in relation to bap intake and PD, employing MR. Utilizing weighted median, MR-Egger, and inverse variance weighting methods, we ensure rigorous causality assessments, meticulously mitigating pleiotropy and heterogeneity biases to uphold finding validity.

**Results:**

Our findings indicate red wine (OR: 1.031; 95% CI 1.001–1.062; *p* = 0.044) and dried fruit consumption (OR: 2.019; 95% CI 1.052–3.875; *p* = 0.035) correlate with increased PD risk, whereas broad beans (OR: 0.967; 95% CI 0.939–0.996; *p* = 0.024) and bap intake (OR: 0.922; 95% CI 0.860–0.989; *p* = 0.023) show protective effects against PD. Employing MR, specifically the IVW method, revealed a significant inverse association between bap intake and gut microbiota, marked by an 8.010-fold decrease in *Parabacteroides goldsteinii* per standard deviation increase in bap intake (95% CI 1.005–63.818, *p* = 0.049). Furthermore, a connection between PD and *Parabacteroides goldsteinii* was observed (OR: 0.810; 95% CI 0.768–0.999; *p* = 0.049), suggesting a potential microbiota-mediated pathway in PD etiology.

**Conclusion:**

Our study links dietary habits to PD risk, showing higher PD risk with red wine and dried fruit consumption, and a protective effect from broad beans and bap. Using MR, we found bap intake inversely correlates with *Parabacteroides goldsteinii* in the gut, suggesting bap influences microbiota. Further, higher *Parabacteroides goldsteinii* levels correlate with lower PD risk, highlighting a complex interplay of diet, gut microbiome, and neurological health. These insights shed light on potential dietary interventions for PD.

## 1 Introduction

Parkinson’s disease (PD), a prevalent long-term condition, poses a significant global health concern (1). In recent years, there has been a significant rise in the prevalence of PD. The objective of treating PD is to reduce symptoms and the likelihood of negative consequences (2). The surge in individuals seeking medical attention has led to a substantial healthcare strain on a worldwide scale (3). In developed countries, individual PD patients bear a financial burden ranging from several thousand to tens of thousands of dollars per year (4). In underdeveloped nations, individuals suffering from PD encounter not only economic hardships but also an increased likelihood of unfavorable consequences as a result of restricted availability of suitable medical care. The development and progression of PD, a neurodegenerative condition marked by the decline of brain cells that produce dopamine, may also be influenced by dietary factors (5). New research has presented findings indicating that nutritional elements might play a role in the emergence and advancement of PD. A thorough examination indicated that adhering to a Mediterranean eating style, which is characterized by a substantial intake of fruits, veggies, and fish, and a limited intake of meat and dairy products, was associated with a decreased occurrence of PD (6). Moreover, a research carried out among the Chinese community in Singapore revealed that individuals who were presently smoking and had increased consumption of caffeine, specifically from black tea, exhibited a decreased likelihood of developing PD (7). Furthermore, the low-carbohydrate, high-fat ketogenic diet has shown promise in providing neuroprotective benefits and improving mitochondrial function in neurodegenerative disorders like PD (8). On the other hand, a different research discovered that consuming more overall fat was linked to a higher likelihood of PD (9). Furthermore, a meta-analysis demonstrated that a greater overall dietary quality was linked to a decreased likelihood of PD (10). These results underscore the potential significance of dietary elements in the prevention and treatment of PD. Nevertheless, additional investigation is required to gain a deeper comprehension of the precise mechanisms and establish focused dietary interventions for this degenerative condition of the nervous system. The investigation of causal relationships is made possible by MR, a robust research technique that employs genetic variants as instrumental variables (IVs). In comparison to alternative methods, MR provides unique benefits when examining the influence of dietary factors on PD. Although there is potential, there is a scarcity of MR studies investigating the causal connections between dietary factors and PD. In order to fill this research void and broaden the current body of knowledge, we performed a comprehensive MR analysis to investigate the intricate connections among different dietary elements and the progression of PD. Our study aims to gain valuable insights into the potential causal connections between diet and PD by using genetic variants as instrumental variables. This will illuminate the underlying mechanisms and guide future interventions. However, based on general knowledge about PD, which is a neurodegenerative disorder characterized by motor symptoms such as tremors, rigidity, bradykinesia, and postural instability, some recent studies suggest that dysregulation of the gut microbiota may play a role in the pathogenesis of PD.

## 2 Methods

MR relies on a number of underlying assumptions. The primary dependence is on the robust correlation between instrumental variables (IVs) and the exposure factors being studied. Additionally, it presupposes that the independent variables are not directly linked to the desired outcome. Finally, it presupposes that the IVs are unrelated to any potential confounding variables that could skew the findings. For this investigation, we employed GWAS summary-level data acquired from the IEU open GWAS initiative. The MRC Integrative Epidemiology Unit (IEU) at the University of Bristol provided support for this project, which involved the curation and analysis of GWAS data from different sources such as the UK Biobank, published articles, and the FinnGen biobank. It is worth mentioning that ethical approval was not necessary for this study since the data used was publicly accessible, anonymized, and de-identified, guaranteeing the safeguarding of participant privacy and confidentiality.

### 2.1 Data sources

**Figure 1:**
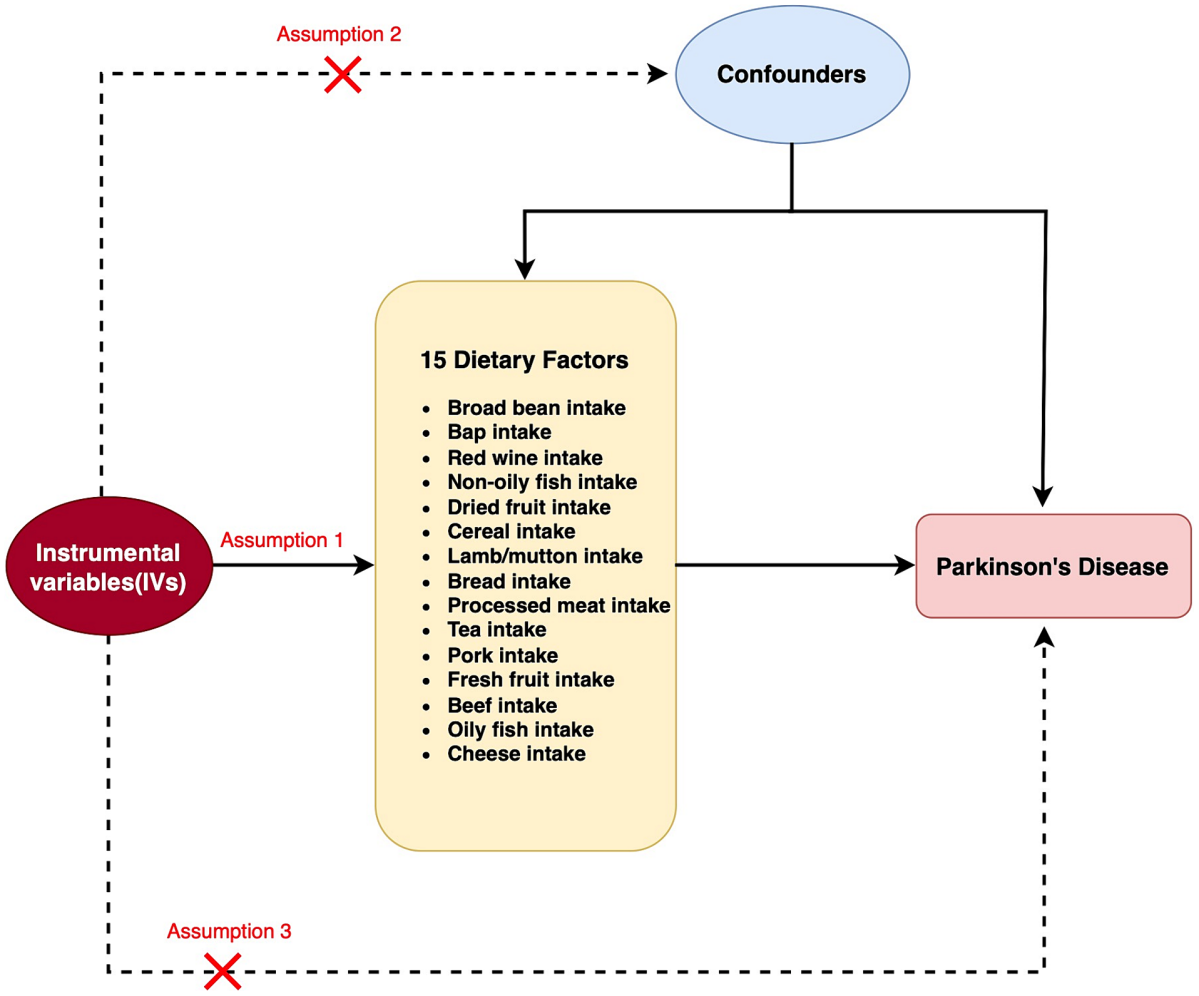
Flow chart for two Mendelian randomization (MR) study for 15 dietary factors and Parkinson’s disease (PD). A node represents a variable, and an arrow represents a direct causal effect.

**Figure 2:**
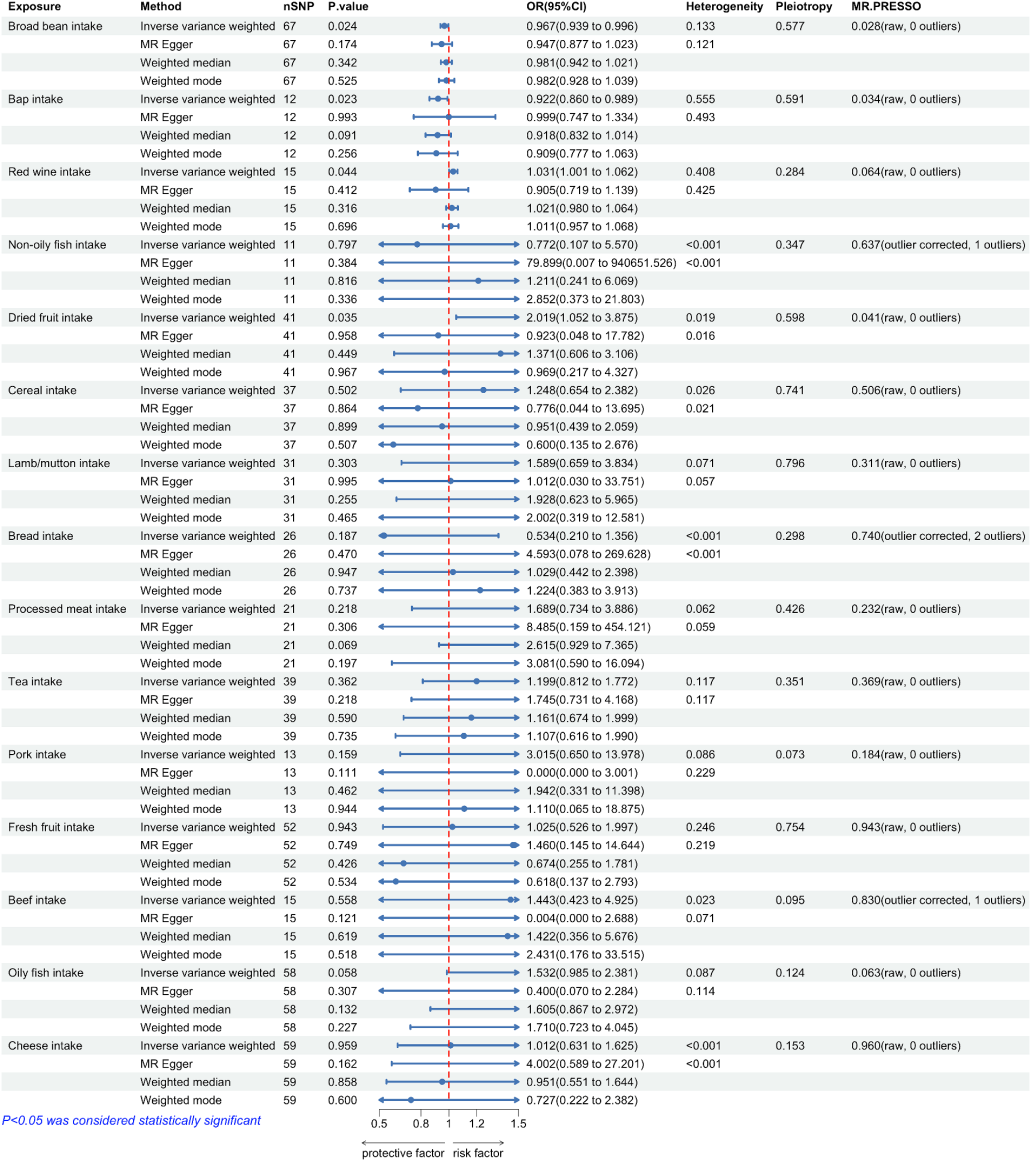
Forest plots summarizing the causal effects, heterogeneity, pleiotropy and MR-PRESSO result of 15 dietary factors and Parkinson’s disease (PD). SNP, single nucleotide polymorphism; OR, odds ratio; CI, confidence interval.

This study investigated a wide array of factors related to diet exposure, encompassing the consumption of meat (processed meat, poultry, beef, non-oily fish, oily fish, pork, and lamb/mutton), staple foods (bread and bap), baps are soft white buns that are excellent for sandwiches, well worth making from scratch. Beverages (tea), fruits (dried and fresh), and other food items (cheese). The analysis also incorporated the relationship between *Parabacteroides goldsteinii*, bap intake, and PD. *Parabacteroides goldsteinii Genus* (Kingdom: Bacteria; Phylum: Bacteroidetes; Class: Bacteroidia; Order: Bacteroidales; Family: Porphyromonadaceae; Genus: Parabacteroides; Species: *Parabacteroides goldsteinii*). The data used in this analysis were acquired from the IEU open GWAS project, which obtained the information either directly or indirectly from the UK Biobank. However, the summary-level data for Parkinson’s disease GWAS were obtained from the International Parkinson’s Disease Genomics Consortium through the IEU open GWAS project. In case the resulting dataset lacks single nucleotide polymorphisms (SNPs) of exposures, we employed SNP proxies as a substitute. For more information about the exposure and outcome datasets, Figures 1, 2 and Supplementary Tables S1, S2 for a comprehensive overview.

### 2.2 The selection of IVs

IVs are essential in MR analysis for establishing causal relationships between exposure factors and outcomes. The IVs usually consist of genetic alterations, with SNPs being the most frequently employed. We acquired genetic variations linked to dietary factors and gut microbiota from the IEU open GWAS project, which is a thorough repository of genetic associations available at https://gwas.mrcieu.ac.uk/ in our research. In order to guarantee the dependability of our analysis, we applied rigorous standards for SNP choice, which encompassed a genome-wide significance threshold (*p* < 5 × 10^−8^), a clumping window exceeding 10,000 kb and a minimal level of linkage disequilibrium (*r*^2^ < 0.001). To obtain more comprehensive details regarding the chosen SNPs (Supplementary Tables S1, S2). In order to establish a solid correlation between independent variables (IVs) and exposure, we employed the F statistic, which is typically regarded as a reliable indicator of a strong association when its value exceeds 10 (7).

### 2.3 Statistical analysis

The primary approach utilized in our research was the IVW method, which employs inverse variance weighting to determine the causal impact. The strong capability of this approach in identifying causation in two-sample MR analysis (11) is widely recognized. Additionally, we compared the outcomes acquired from the IVW technique with those derived from the Weighted Median and MR-Egger approaches. The weighted median approach permits up to 50% of the IVs to be invalid, whereas the MR-Egger method allows for the possibility of all IVs being invalid. Hence, when all three models produce congruent outcomes, it offers more persuasive proof. In order to evaluate diversity in the IVW model, we employed Cochran’s *Q*-test, deeming diversity to exist if the *p*-value is below 0.05. Nevertheless, it is crucial to acknowledge that the existence of diversity does not automatically invalidate the IVW model. Furthermore, we employed the MR-Egger approach, which permits non-zero intercepts, for the identification of directional pleiotropy. To assess the impact of individual SNPs on the results, we performed a leave-one-out analysis. In this analysis, each SNP was sequentially removed and the analysis was repeated. The MR-PRESSO method was used to identify outliers, which were then excluded from the analysis. After excluding the outliers, the MR analysis was repeated. Statistical power for our MR analyses was estimated using an online calculator available at https://sb452.shinyapps.io/power. The R version 4.0.3 software was employed, along with the “TwosampleMR” and “MR-PRESSO” packages, to conduct all the analyses (12).

### 2.4 Mediation analysis

The potential impact of dietary factors on disease may be related to the gut microbiota. In this study, we first used casual dietary factors as exposures and conducted two-sample MR analyses with the gut microbiota. Subsequently, gut microbiota that showed causal associations with casual dietary factors were used as exposures in two-sample Mendelian randomization analyses with PD, aiming to evaluate the mediating role of the gut microbiota in the relationship between casual dietary factors and PD.

## 3 Results

The correlation between dietary elements was examined by analyzing 15 distinct exposure factors. As shown in Figure 1, a flow chart presents the two-sample study design for 15 dietary factors and PD. The research encompassed a variety of 11–67 (SNPs). All factors had F-statistics greater than 30, with values ranging from 33.556 to 155.282. Individuals of European descent were included in the study, with sample sizes ranging from 275,160 to 11,4,497,213, with little overlap between exposures and outcomes. Figure 2 contains additional details about the exposures and outcomes. The number of SNPs utilized for various exposures varied from 11 to 67, and subsequent removal of outliers resulted in a range of 5 to 90 (with the identification of 0 to 2 outliers in different exposures). All of our MR estimates had a statistical power of at least 95%. Figure 2 contains additional details about the exposures and outcomes. Our analysis demonstrates a strong correlation between the instrumental variables (IVs) used in our study and the exposures, as indicated by the F statistic (excluding anomalies) ranging from 33.556 to 155.282. The study identified a total of four casualties (*p* < 0.05 using the IVW method). An elevated risk of PD was observed with the consumption of Red wine (OR: 1.031; 95% CI: 1.001–1.062; *p* = 0.044) and Dried fruit (OR: 2.019; 95% CI: 1.052–3.875; *p* = 0.035). The connection between consumption of red wine was additionally verified through the MR-Egger (after excluding outliers OR: 0.905; 95% CI: 0.719–1.139; *p* = 0.412) and weighted median (after excluding outliers OR: 1.021; 95% CI: 0.98–1.064; *p* = 0.316) approaches. Additionally, the connection between consumption of dehydrated fruit was additionally reinforced by the MR-Egger (after eliminating anomalies OR: 0.923; 95% CI: 0.048–17.782; *p* = 0.958) and weighted median (after eliminating anomalies OR: 1.371; 95% CI: 0.606–3.106; *p* = 0.449) models. Conversely, the consumption of broad beans (OR: 0.967; 95% CI: 0.939–0.996; *p* = 0.024) and bap (OR: 0.922; 95% CI: 0.86–0.989; *p* = 0.023) exhibited a beneficial impact. However, the MR-Egger model did not yield any significant findings (*p* > 0.05) for the consumption of broad beans (OR: 0.947; 95% CI: 0.877–1.023; *p* = 0.174) and bap (OR: 0.999; 95% CI: 0.747–1.334; *p* = 0.993). In the Weighted median model, the OR for broad bean intake was 0.981 (95% CI: 0.942–1.021; *p* = 0.342), and for bap intake it was 0.918 (95% CI: 0.832–1.014; *p* = 0.091). This study also revealed that the intake of non-oily fish had no significant effect (OR: 0.772; 95% CI: 0.107–5.57; *p* = 0.797; Outliers excluded OR: 1.50; 95% CI: 0.295–7.603; *p* = 0.637), as well as cereal intake (OR: 1.248; 95% CI: 0.654–2.382; *p* = 0.502; No outliers), lamb/mutton intake (OR: 1.598; 95% CI: 0.659–2.251; *p* = 3.834; No outliers), bread Intake (OR: 0.543; 95% CI: 0.21–1.356; *p* = 0.187; Outliers excluded OR: 0.873; 95% CI: 0.394–1.932; *p* = 0.740), processed meat intake (OR: 1.689; 95% CI: 0.734–3.886; *p* = 0.218; No outliers), tea intake (OR: 1.199; 95% CI: 0.812–1.772; *p* = 0.362; No outliers), and pork intake (OR: 3.015; 95% CI: 0.650–13.978; *p* = 0.184; No outliers). The odds ratio for fresh fruit intake was 1.025 (95% CI: 0.526–1.997; *p* = 0.943) with no outliers. For beef intake, the odds ratio was 1.443 (95% CI: 0.423–4.925; *p* = 0.568) with outliers excluded. Additionally, the odds ratio for beef intake was 0.892 (95% CI: 0.329–2.484; *p* = 0.830) with outliers excluded. Oily fish intake had an odds ratio of 1.532 (95% CI: 0.985–2.381; *p* = 0.058) with outliers excluded. Lastly, the odds ratio for cheese intake was 1.012 (95% CI: 0.631–1.625; *p* = 0.959) with no outliers.

**Figure 3:**
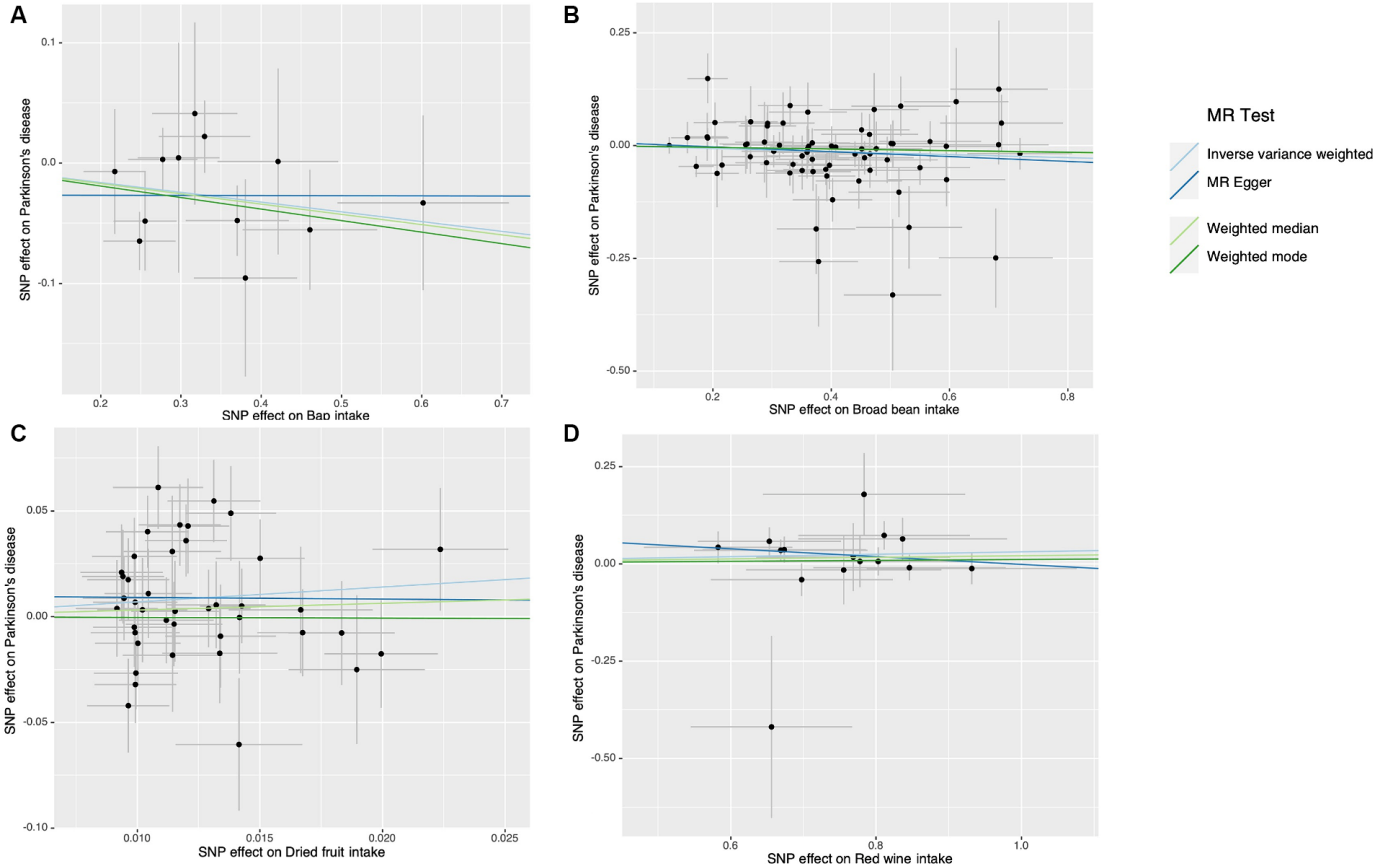
Scatter plots of the causal effect of dietary factors on Parkinson’s disease (PD). **(A)** Bap intake on PD. **(B)** Broad been intake on PD. **(C)** Dried fruit intake on PD. **(D)** Red wine intake on PD.

**Figure 4:**
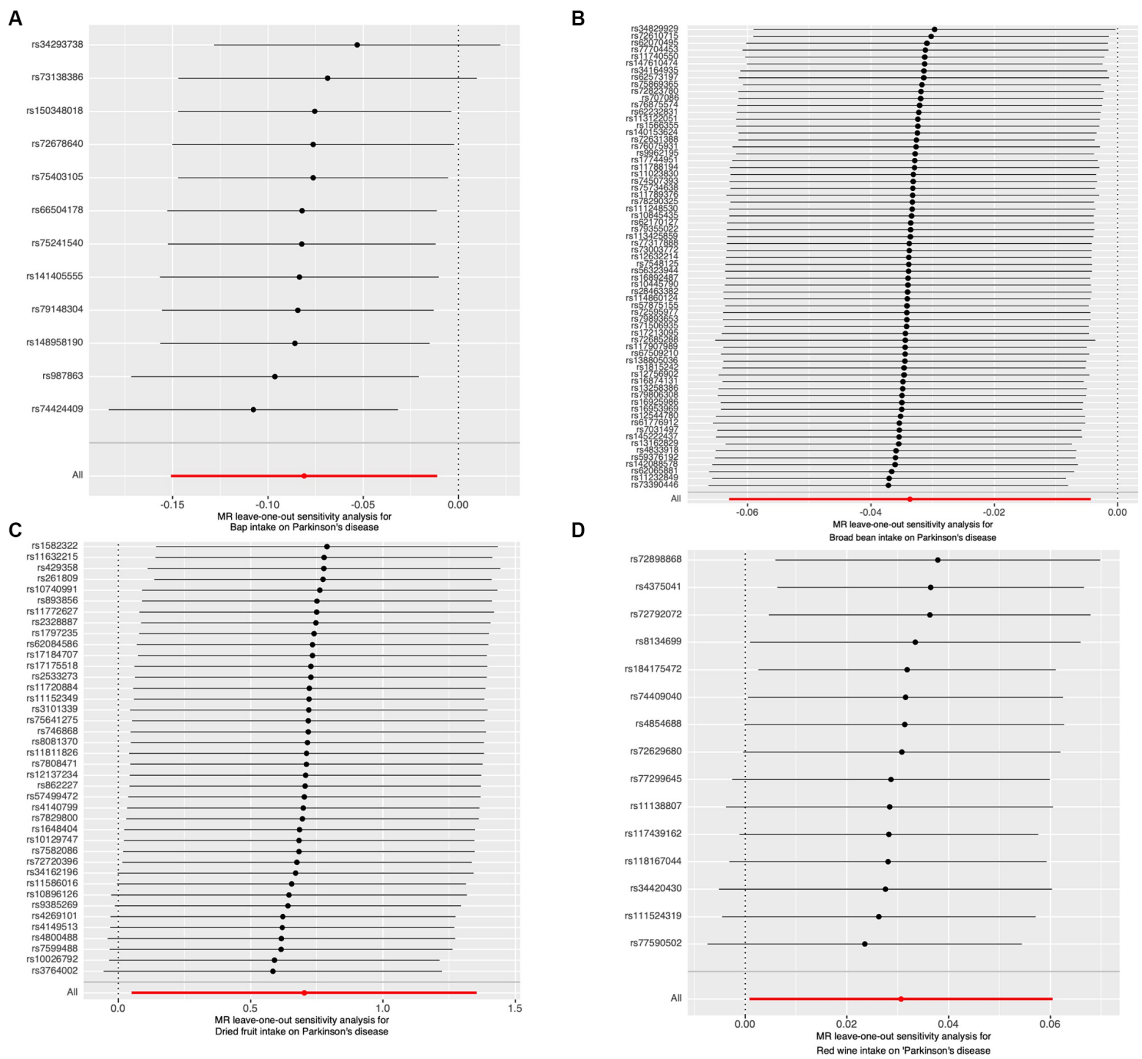
The results of Leave-one-out analyses. **(A)** Bap intake. **(B)** Broad been intake frequency. **(C)** Dried fruit intake. **(D)** Red wine intake.

**Figure 5:**
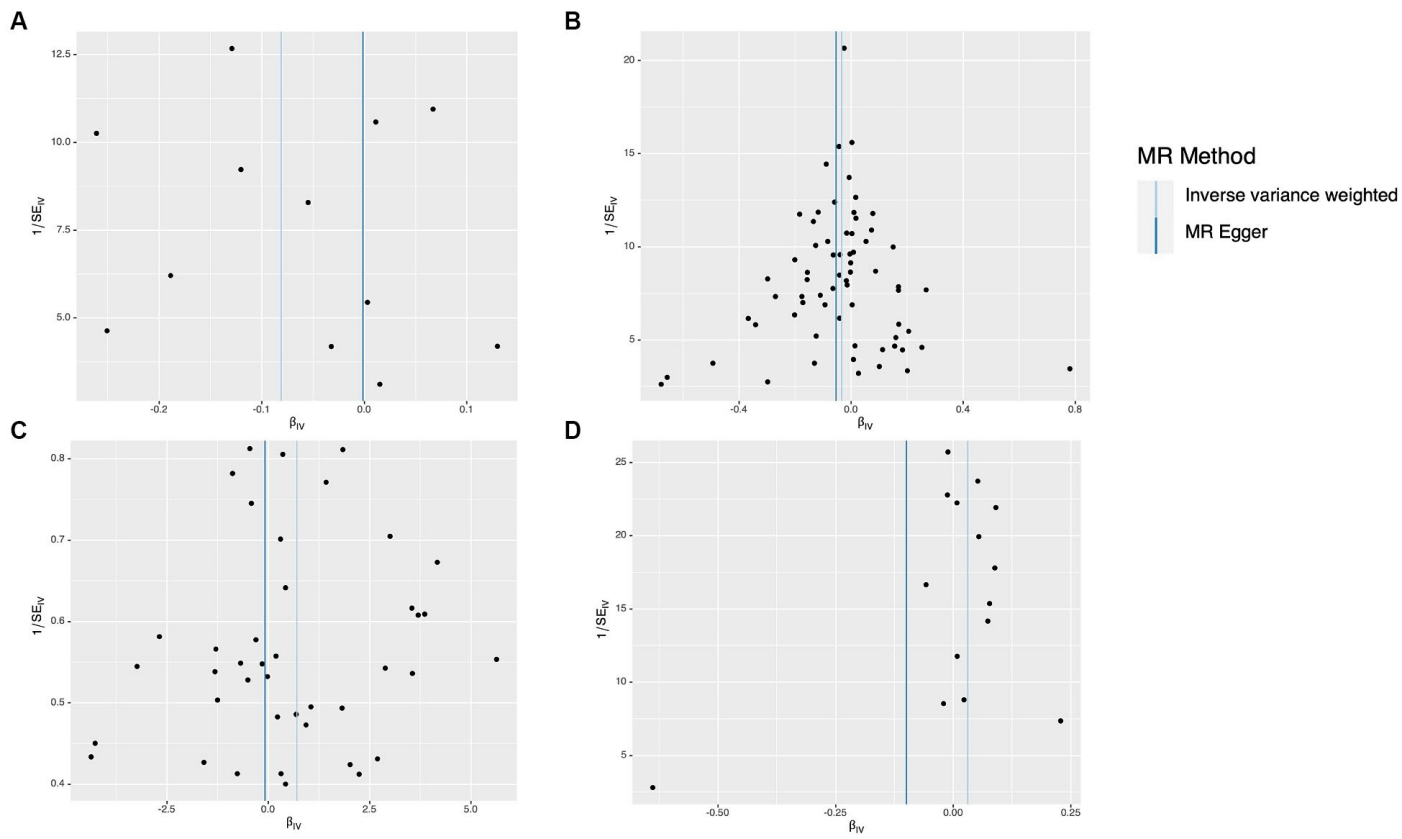
Funnel plots for dietary factors and Parkinson’s disease (PD). The MR estimate for each SNP is plotted against its minor-allele frequency corrected association with bap intake **(A)**, broad been intake **(B)**, dried fruit intake **(C)**, red wine intake **(D)**. The vertical lines show the results of IVW or MR-ER using all SNPs. IVW, inverse variance weighted; MR, Mendelian randomization; SNP, ingle nucleotide polymorphism.

The results of the study did not indicate any correlation with PD, both prior to and subsequent to removing outliers. Figure 2 displays the findings. Scatter plots of the causal effect of dietary factors on PD: (A) bap intake, (B) Broad bean intake, (C) Dried fruit intake, (D) Red wine intake. Despite the presence of diversity in various exposures (as indicated by Cochrane’s *Q*-test, *p* < 0.05), the MR-Egger intercept analysis did not provide any indication of directional pleiotropy (Figure 3). The results of Leave-one-out analyses: (A) bap intake, (B) broad bean intake frequency, (C) dried fruit intake, (D) red wine intake. Funnel plots for dietary factors and PD. The strength of the affirmative findings was validated by conducting leave-one-out analysis. Although we do not have a single SNP with a discernible effect on the outcome when considering individual exposure, our leave-one-out plot demonstrates a commendable level of stability across the population SNPs. This observation implies that the influence of population SNPs exposures on the outcome is resilient to idiosyncratic anomalies associated with any particular SNPs (Figure 4). The MR estimate for each SNP is plotted against its minor-allele frequency corrected association with bap intake (A), broad bean intake (B), dried fruit intake (C), red wine intake (D). The vertical lines show the results of IVW or MR-ER using all SNPs. IVW: inverse variance weighted; MR: Mendelian randomization; SNP, single nucleotide polymorphism. Funnel plots for dietary factors and PD (Figure 5). Consistently, the findings from the MR-PRESSO examination were in agreement with the outcomes of the IVW model, uncovering causal associations solely in the consumption of Broad beans, bap, red wine, and dried fruits (Figure 2).

**Figure 6:**
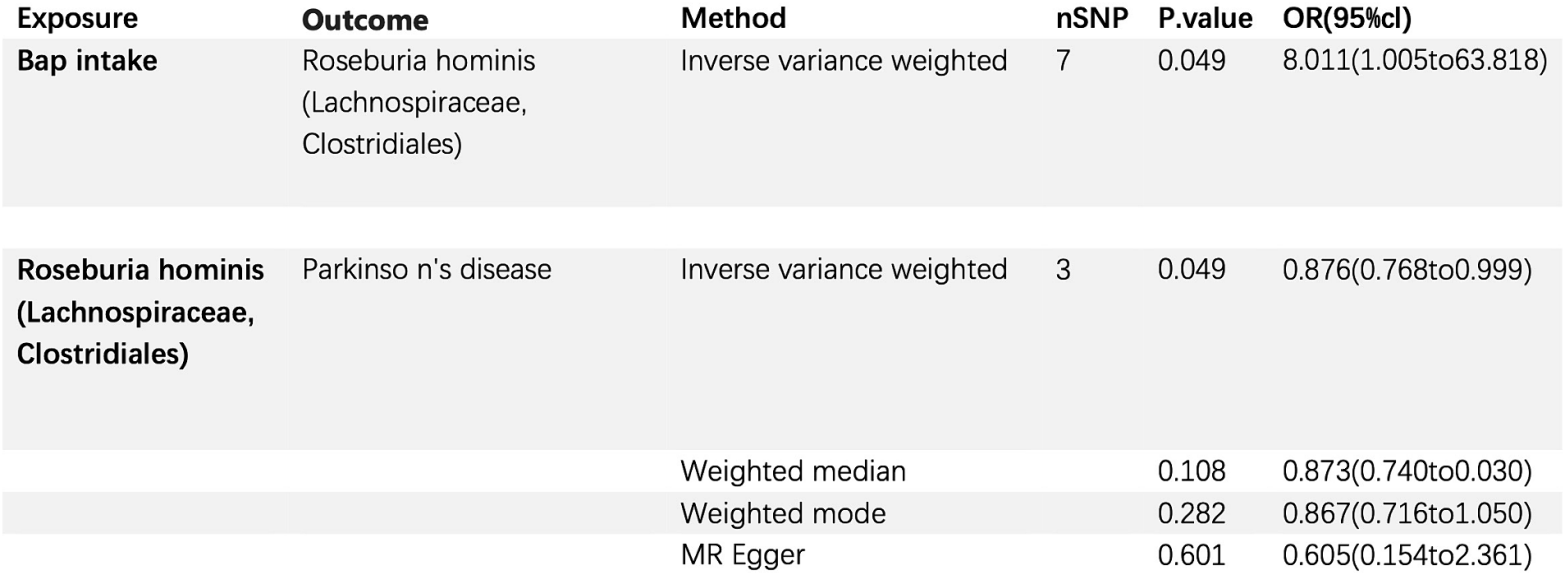
Bap intake, Parkinson’s disease, and the relationship with gut commensal bacterium *Parabacteroides goldsteinii* (Family: Porphyromonadaceae; Order: Bacteroidales).

A MR study was conducted to examine the relationship between bap intake and the gut commensal bacterium *Parabacteroides goldsteinii*. Bap intake served as the exposure variable, and the analysis was performed using the inverse variance weighting (IVW) method. The investigation involved seven single nucleotide polymorphisms (SNPs) with a collective *p*-value of 0.049. The odds ratio (OR) for the association between PD and *Parabacteroides goldsteinii*. Abundance was calculated as 8.011 (95% CI: 1.005–63.818). This suggests that elevated bap intake may modulate the population size of *Parabacteroides goldsteinii* in the gut microbiota. Notably, *Parabacteroides goldsteinii* appears to exhibit a protective effect against PD development (Figure 6).

However, it is crucial to acknowledge the inherent limitations of MR analyses, which rely on assumptions about the validity of genetic instruments and potential.

unaccounted pleiotropic effects. Consequently, these findings should be interpreted with caution and require further corroboration through independent studies using complementary methodologies.

## 4 Discussion

The main discovery of this analysis using MR is the correlation between consumption of red wine, broad bean, bap, and dried fruit with PD. While previous MR investigations have explored the risk or protective elements of PD (13–15), there is limited research on the correlation between consumption of meat, staple foods, fruits and vegetables, and beverages in relation to PD. Our analysis addresses this gap by considering anomalies through a second MR analysis and utilizing updated outcomes from the IVW model to establish causality. PD is a multifaceted genetic disorder that is affected by both lifestyle choices and environmental elements, alongside a range of genetic variations (16–19). Research suggests that exposure to pesticides (20, 21) and a history of melanoma (22) are associated with an increased risk of developing PD, while alcohol consumption may offer some level of protection. PD has a significant global economic burden and greatly affects the quality of life of patients (23–25). Our study findings can assist clinicians in enhancing health education for PD patients and promoting dietary changes, such as reducing red wine and dried fruit intake while increasing broad bean and bap intake. Individuals who are at a high risk of developing PD can also benefit from making these dietary changes. In general, this research enhances our comprehension of the hazards and safeguarding elements linked to PD.

Numerous epidemiological investigations have explored the possible correlation between the intake of red wine and neurodegenerative disorders, including PD and Alzheimer’s disease (AD). A study found that consuming red wine is linked to a reduced likelihood of age-related neurodegenerative disorders, particularly PD (26). Moreover, studies have revealed that resveratrol, a type of polyphenol present in red wine, can augment the elimination of amyloid-beta peptides, which are recognized for their involvement in the progression of AD pathology (27, 28). The antioxidative characteristics of red wine, mainly attributed to the existence of polyphenols like resveratrol (29, 30), are responsible for its neuroprotective benefits. In our research, we discovered that the intake of red wine can be considered a contributing factor to PD. In contrast to observational research, MR investigations employ genetic variations, specifically single nucleotide polymorphisms (SNPs), as instrumental variables in order to reduce the impact of confounding factors and reverse causation. Several factors can account for the differences observed between MR studies and observational studies in relation to the connection between PD and red wine consumption. Firstly, even after adjusting for potential confounding variables, observational studies may still be impacted by unmeasured confounders. Furthermore, the MR analysis did not discover a U-shaped correlation between consumption of red wine and PD. Hence, it is imperative to conduct further research by incorporating supplementary observational studies and pioneering MR studies in order to gain deeper insights into the correlation between the consumption of red wine and PD. The results of our study indicate that consuming red wine is linked to a higher likelihood of developing PD, and we hypothesize that there may be a positive association between the Red wine intake and the risk of PD. There has not been a thorough investigation into the correlation between consumption of dehydrated fruits and PD. Nevertheless, the study did not specifically examine the influence of consuming dried fruit on the risk of PD. Despite the potential health advantages associated with dried fruit consumption, such as decreased cancer risk and enhanced cardiovascular health (5, 31), there is a lack of dependable epidemiological evaluations specifically concerning the connection between dried fruit intake and PD (32). Hence, additional investigation is required to explore the possible impacts of consuming dehydrated fruits on both the susceptibility and treatment of PD.

Broad bean intake and bap intake have been identified as potential protective factors. While the literature provided does not explore the precise correlation between the consumption of broad beans, bap intake, and PD, it does contain research on the connection between various dietary elements and the likelihood of developing PD. For example, research revealed an inverse relationship between the intake of coffee and caffeine and the likelihood of PD (33). Another investigation indicated that consuming a diet abundant in fruits, veggies, legumes, whole grains, poultry, and fish was associated with a reduced risk of PD (34). A different meta-analysis indicated that adhering to a Mediterranean eating style, which involves consuming ample amounts of fruits, vegetables, and fish, was linked to a decreased occurrence of PD (5). Nonetheless, this study did not find any proof of a cause-and-effect connection between the consumption of meat or poultry and PD. While the precise correlation between consumption of broad beans and bap and PD remains uncertain, these studies highlight the possible influence of dietary elements on the risk and control of PD. Additional investigation is required to analyze the impact of consuming broad beans on PD. Gaining a comprehensive comprehension of the correlation between MR and randomized controlled trials (RCTs) is of utmost importance. MR serves as an effective approach to mitigate confounding bias (35). Nevertheless, it is crucial to acknowledge that although MR offers a beneficial supplement to RCTs, it must not be regarded as a replacement for them. Therefore, caution must be exercised when interpreting this conclusion. The study also elucidates the underlying mechanism linking bap intake with PD, revealing that bap consumption exerts an influence on the gut microbe Parabacteroides *Parabacteroides goldsteinii* which itself serves as a protective factor against PD development. This finding substantiates the observed association between bap intake and PD risk, establishing a plausible causal pathway mediated through alterations in gut microbial composition.

The complete understanding of how dietary factors impact PD is still lacking. The gut microbiome offers a potential route. The consumption of various foods can impact the microbiota composition in the gastrointestinal tract, consequently influencing nutrient metabolism (36). Numerous investigations have examined the correlation between PD and the microbial community residing in the gastrointestinal tract. A research study examined the makeup and variety of gut bacteria in individuals diagnosed with AD and discovered notable distinctions when compared to the control group (37). While the primary focus of this study was on AD, it implies that changes in the gut microbiota could potentially have a connection to neurodegenerative conditions. A different investigation analyzed the long-term consistency of gut microbiota in individuals with PD and its connection to the advancement of the disease (38), emphasizing the necessity for additional studies on the gut microbiota in PD, especially in individuals who are newly diagnosed. In future research, it is crucial to ensure comparability and utility, as highlighted by a review that summarized studies on the gut microbiome in PD (39). Furthermore, multiple research studies have indicated a connection between the microbial composition of the gastrointestinal tract and neurodegenerative disorders, particularly Parkinson’s disease (40). An imbalance in the gut microbiota can affect the development of neurodegenerative diseases, as it has been linked to the regulation of brain activity and cognitive function (41). Inflammation and bacterial translocation are associated with dysregulation of the gut microbiota in PD patients. The connection between imbalanced gut bacteria and the development of PD has been examined, and possible treatment choices have been emphasized (42). Taken together, these discoveries indicate a possible link between the intestinal microbiota and PD. Nevertheless, additional investigation is required to fully grasp the fundamental processes and create specific interventions.

Caution should be exercised when interpreting the findings of the MR analysis, which suggested possible links between the consumption of red wine, dried fruit, broad beans, and bap with PD. The causality identified by the MR analysis primarily represents the impacts of prolonged exposure to these factors, while short-term exposures may lack clinical significance. Furthermore, a significant constraint of MR is its incapacity to distinguish causal connections among distinct time intervals. For example, a study using MR showed a direct connection between vitamin D and multiple sclerosis (43). However, this association was only noticeable during childhood or prior (44). Additionally, univariate MR analyses solely uncover general impacts between exposures and outcomes, without capturing the direct impacts between them. The relationship between exposures and outcomes can be exceedingly intricate.

This study possesses both strengths and limitations. MR employs genetic variations as instrumental variables (IVs) to establish causality and tackle concerns such as reverse causality and confounding. In order to guarantee the precision of the MR analysis, sensitivity and pleiotropic analyses were conducted. To reduce potential biases, the research employed European populations from various countries for both exposures and outcomes. Never the less, it is crucial to recognize the constraints of this research. Initially, despite the fact that the instrumental variables employed in this research demonstrated robust connections with the exposure (F-statistics >10), a considerable proportion of the F-statistics were lower than 100, potentially impacting the accuracy of the findings. Additionally, the research was incapable of further classifying distinct forms of food consumption or distinguishing the impacts of different dietary combinations. Unfortunately, the unavailability of summary-level GWAS data for different genders prevented the possibility of conducting a sex-specific analysis.

## 5 Conclusion

The research revealed a correlation between the intake of broad beans and bap and a reduced likelihood of PD. On the other hand, the frequency of consuming Red wine and dried fruit was linked to an increased likelihood of developing PD. Furthermore, the research discovered that there was no notable correlation between the Non-Oily fish, Cereal, Lamb/mutton, Bread, Processed meat, Tea, Pork, Fresh fruit, Beef, Oily fish, Cheese, and gut microbiota with PD. Statement regarding the availability of data This study analyzed datasets that were made available to the public. The data can be located at the following source: The IEU open GWAS project (https://gwas.mrcieu.ac.uk/) contains all the GWAS data utilized in this research.

## Data availability statement

The datasets presented in this study can be found in online repositories. The names of the repository/repositories and accession number(s) can be found in the article/Supplementary material.

## Author contributions

SZ: Conceptualization, Data curation, Formal analysis, Funding acquisition, Investigation, Methodology, Project administration, Resources, Supervision, Validation, Visualization, Writing – original draft, Writing – review & editing. AY: Data curation, Formal analysis, Investigation, Methodology, Project administration, Supervision, Validation, Writing – review & editing. HL: Funding acquisition, Supervision, Writing – review & editing. CZ: Data curation, Validation, Visualization. CY: Data curation, Validation, Visualization.
